# Determining utility values related to malaria and malaria chemoprophylaxis

**DOI:** 10.1186/1475-2875-9-92

**Published:** 2010-04-09

**Authors:** Anne E McCarthy, Doug Coyle

**Affiliations:** 1 Tropical Medicine and International Health Clinic, Division of Infectious Diseases Ottawa Hospital General Campus, University of Ottawa, 501 Smyth Road, Ottawa, Ontario K1H 8L6, Canada; 2 Clinical Epidemiology Program, Ottawa Health Research Institute, Department of Epidemiology and Community Medicine, University of Ottawa, Ottawa, Ontario, Canada

## Abstract

**Background:**

Chemoprophylaxis for travellers' malaria is problematic. Decision modeling may help determine optimal prevention strategies for travellers' malaria. Such models can fully assess effect of drug use and disease on quality of life, and help travellers make informed values based decisions. Such models require utility values reflecting societal preferences over different health states of relevance. To date, there are no published utility values relating to clinical malaria or chemoprophylaxis adverse events.

**Methods:**

Utility estimates for health states related to falciparum malaria, sequelae and drug-related adverse events were obtained using a self-administered visual analogue scale in 20 individuals. Utility values for health states related to clinical malaria were obtained from a survey of 11 malaria experts questioned about length of hospital stay or equivalent disability with simple and severe travellers' malaria.

**Results:**

The general public (potential travellers), were more tolerant of taking prophylaxis if associated with no or mild AEs and least tolerant of mild sequelae from malaria and severe drug related events. The rating value reported for taking no prophylaxis was quite variable. Tropical medicine specialists estimated a mean hospital stay 3.23 days (range 0.5-4.5 days) for simple and 6.36 days (range 4.5 - 7 days) for severe malaria.

**Conclusions:**

This study provides a benchmark for important utility value estimates for modeling malaria and drug-related outcomes in non-immune travellers.

## Background

There is little doubt that experiencing clinical malaria or drug-related adverse events (AEs) with malaria chemoprophylaxis will impact an individual's quality of life. Currently, there is disagreement, and often much heated debate among travel medicine providers and public health authorities concerning the benefit of taking chemoprophylaxis to prevent clinical malaria within certain areas of risk [1-10]. Those advocating for decreasing use of chemoprophylaxis cite drug related AE and risk-benefit assessments.

To best make decisions about the appropriateness of malaria chemoprophylaxis, it is necessary to construct a decision-model, which allows a systematic approach to assessing the trade-off between risks and benefits. Such a model would require the use of utility values, which reflect societal preferences over different health states of relevance. Utility values are measures of a decision maker's relative preferences for different outcomes associated with health care interventions. When using utility values, analysts place a value of a health outcome assessing the outcome relative to death, which has a score of 0 and perfect health which has a score of 1. By aggregating utility values weighted by the probability of health outcomes an aggregate score summarizing the value of an intervention can be obtained.

However, to date, there are no published utility values relating to clinical malaria or chemoprophylaxis adverse events. Such utility values are necessary for the construction of models that would allow full examination of risks and benefits, and could consider all possible outcomes related to malaria and chemoprophylaxis, even severe adverse outcomes and death. An additional benefit to such a model would be the ability to simulate outcomes from hundreds of thousands of travellers to areas with differing risk of malaria and differing use of chemoprophylaxis. Such simulations would provide outcome measures for these travellers, including numbers of resulting malaria cases and deaths with each option, as well as drug related events. Such information would be of benefit to policy makers and travel medicine providers advising travellers about malaria risk and prevention strategies.

Given the lack of available utility values, the objective of this study was to determine the utility values related to clinical malaria and drug- related adverse events, which can be employed in decision models. These estimates were obtained from two anonymous surveys, carried out, after ethics approval (London School of Hygiene and Tropical Medicine and the Ottawa Health Research Ethics Board) and voluntary informed consent.

## Methods

### Survey of general public (Potential travellers)

**Figure 1:**
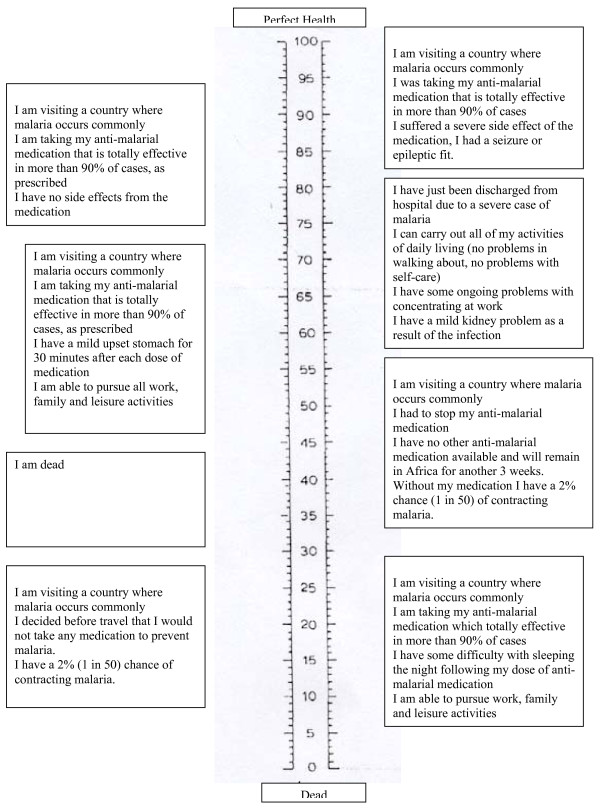
**Malaria Visual Analogue Scale: Risk and Outcomes of Malaria and Chemoprophylaxis**.

There are risks (AEs) and benefits (reduction of clinical malaria) associated with drugs used to prevent malaria. Therefore, it is necessary to weigh the risks against the values of the therapy. Utility estimates for health states related to non-severe falciparum malaria, sequelae and drug-related AEs were obtained through a convenience sample of 20 non-health care employees in an academic hospital setting (14 females and 6 males) using a self-administered visual analogue scale (VAS) based on the scale employed as part of the EQ-5D instrument[11]. The EQ5D is a generic, quality of life instrument developed to elicit an overall measure of health status. The EQ-5D contains both a health status questionnaire and a self-administered visual analog scale (VAS), which was used in this analysis. The scale is a standardized, non-disease specific VAS developed to describe and attach values to a common core of health states detailing various quality of life levels. The scale is organized vertically like a thermometer and eight different descriptions of health states are organized at the periphery of the scale, so that all states can be considered simultaneously. The scale is generally anchored with perfect health as the highest possible value and death or worst imaginable health as the bottom anchor. The 100-millimetre thermometer ranges from 0 (dead) to 100 (perfect health) and participants rate the value of a certain health state by drawing a line from the description text box to intersect the thermometre. The resulting value is the number of millimeters from the zero or "dead", divided by 100. Figure 1 presents the design of the VAS as well as a description of various scenarios related to malaria risk and outcomes, chemoprophylaxis and health states that were assigned a value by each of the respondents.

When a VAS is used to estimate societal preferences, a power calculation has been suggested as a means to convert the values (v) obtained into utilities (u)[12]. This is done by using a standard power function relationship given by: (1-u) = (1-v)^α^, where α allows incorporation of the attitude to risk within the population (alpha > 1 represents risk aversion)[12,13]. A value of alpha of 2.29 was assumed based on a previous Canadian study[13]. The alpha was obtained through comparing individuals' scores from using both a VAS and a standard gamble exercise - a more complex measure of utility measurement[14]. Thus, if a respondent rated a health state at 50, the VAS score would be 0.5 (50/100) and the utility value would be 0.80 (1-(1-0.5)^2.29^).

### Survey of malaria experts

Utility values for clinical malaria health states in travellers were obtained from a brief questionnaire of 11 tropical medicine experts (four females and seven males) each with five or more years of experience treating malaria. They were questioned about length of hospital stay (including days in ICU) or equivalent disability related to simple and severe clinical malaria in a non-immune traveller. Utility values were calculated for a one- week time of risk. This timeframe was chosen since travellers itineraries vary widely, and having a weekly value may be more easily applicable within a model that addresses variable length of travellers malaria risk. As per previous studies, conservative assumptions were adopted relating to the utility value associated with the duration of hospitalization (LOS) - a utility value of zero during length of stay[15]. So that a one week utility value (u) is defined as follows:

u = 1 - (LOS/7). Thus, if a respondent felt that severe clinical malaria required a five-day hospitalization the derived utility value was 0.29 (1 - 5/7).

## Results

### Survey of general public

The results of the malaria and malaria drug VAS and their respective utility estimates are presented in descending order of value in Table 1. The pooled value for mild drug-related adverse events was calculated by combining the results of values for mild gastrointestinal and mild sleep related AEs. A pooled result for the value of being on no prophylaxis was calculated by combining the value of stopping chemoprophylaxis due to adverse events while traveling with values for taking no prophylaxis despite malaria risk. For the utility value estimates of mild drug-related AE and being off prophylaxis, only the pooled results were used, since those values would be required for modeling purposes.

**Table 1:** Survey Results and Utility Estimates

General Public/Potential Travellers (N = 20)				
	**Survey Results**		**Utility Values**	

**VAS Scenario**	**Mean**	**Standard Error**	**Mean**	**Standard Error**

On Drug No AE	0.9725	0.0064	0.9993	0.0003

Pooled Mild AE	0.8555	0.0125	0.9802	0.0035
Mild Gastrointestinal AE	0.8930	0.012		
Mild Sleep AE	0.8180	0.0186		

Pooled Off Drug	0.5368	0.0400	0.7541	0.0430
Stopped drug	0.5325	0.0547		
No drug	0.5410	0.0597		

Mild Malaria Sequelae (RF)	0.3920	0.0448	0.6304	0.0583

Severe Drug Related AE	0.3165	0.0323	0.5363	0.0555

**Malaria Experts (N = 11)**				

	**Survey Results (days)**		**Utility Values**	

	**Mean**	**Standard Error**	**Mean**	**Standard Error**

Simple Malaria	3.23	0.2727	0.5390	0.0390

Severe Malaria	6.36	0.426	0.0910	0.0610

### Survey of malaria experts

The 11 tropical medicine specialists estimated a mean duration of hospitalization or equivalent disability for simple malaria of 3.23 days (range 0.5-4.5 days). For severe or complicated malaria the estimated mean length of stay was 6.36 days (range 4.5 - 7 days). The utility estimates calculated from the questionnaire results are presented in Table 1.

## Discussion and Conclusions

The results from members of the general public, who represent potential travellers, suggests that they are more tolerant of taking prophylaxis whether associated with no or mild AEs. They are least tolerant of mild malaria sequelae and severe drug- related AEs. The rating value reported for being off prophylaxis was quite variable. This may in part reflect the scenario presented to participants related to malaria risk and efficacy of chemoprophylaxis communicated in the VAS research tool. An individual traveller who is unaware or unconcerned about malaria risk related to their travel may provide different results. Therefore, one limitation of this tool may be the participants' perceived malaria risk (our scenario included the risk for travel to West Africa), the results may vary with lower areas of risk. The survey respondents were not a random sample, which may limit the generalizability of the results obtained. However, these estimates are still the best available estimates in the literature with respect to utility values for malaria and malaria chemoprophylaxis related health states. The excess representation of women (70%) may also impact our results. As well, the authors recognize that the tolerance of malaria and drug-related outcomes may change in different traveling populations. In future the methods adopted in this study can be used in different populations with varying malaria risk and tolerance, to ensure generalizability.

The estimated days of hospital stay for complicated and non-complicated malaria provided by the clinical experts are in keeping with previous case series and observational studies of outcomes of imported travellers' malaria in non-endemic countries. Those studies reported hospital stays of 3-4 days (range 0-8) for simple and 5-9 days (range 2-20) for severe PF malaria[16-19]. It is possible that the experience of the experts used for the survey, mainly from North America, may differ from providers in other non-endemic areas.

In conclusion, this study provides a benchmark for important utility value estimates for those modeling malaria and drug-related outcomes in non-immune travellers. Further studies can be conducted using random samples of the general public and a larger sample size, but given the current lack of data, it could be suggested that these estimates can be used when assessing the risk benefit trade off associated with malaria chemoprophylaxis.

## Competing interests

The authors declare that they have no competing interests.

## Authors' contributions

Both authors have had significant and continued intellectual contribution to this research and manuscript preparation. AM conceived of, designed and carried out this study with input from DC. Both authors carried out the analysis and interpretation of the data. AM prepared the initial draft of the manuscript and DC provided revisions. Both authors have approved the final version of the manuscript, submitted here for publication.
